# Control of Carbon Assimilation and Partitioning by Jasmonate: An Accounting of Growth–Defense Tradeoffs

**DOI:** 10.3390/plants5010007

**Published:** 2016-01-15

**Authors:** Nathan E. Havko, Ian T. Major, Jeremy B. Jewell, Elham Attaran, John Browse, Gregg A. Howe

**Affiliations:** 1 Institute of Biological Chemistry, Washington State University, Pullman, WA 99164, USA; nhavko@vetmed.wsu.edu (N.E.H.); jbjewell@wsu.edu (J.B.J.); elham.attaran@wsu.edu (E.A.); 2 Department of Energy-Plant Research Laboratory and Department of Biochemistry and Molecular Biology, Michigan State University, East Lansing, MI 48824, USA; majori@msu.edu

**Keywords:** jasmonate, growth–defense tradeoff, defense response, growth inhibition, leaf mass per area, carbon partitioning

## Abstract

Plant growth is often constrained by the limited availability of resources in the microenvironment. Despite the continuous threat of attack from insect herbivores and pathogens, investment in defense represents a lost opportunity to expand photosynthetic capacity in leaves and absorption of nutrients and water by roots. To mitigate the metabolic expenditure on defense, plants have evolved inducible defense strategies. The plant hormone jasmonate (JA) is a key regulator of many inducible defenses. Synthesis of JA in response to perceived danger leads to the deployment of a variety of defensive structures and compounds, along with a potent inhibition of growth. Genetic studies have established an important role for JA in mediating tradeoffs between growth and defense. However, several gaps remain in understanding of how JA signaling inhibits growth, either through direct transcriptional control of JA-response genes or crosstalk with other signaling pathways. Here, we highlight recent progress in uncovering the role of JA in controlling growth-defense balance and its relationship to resource acquisition and allocation. We also discuss tradeoffs in the context of the ability of JA to promote increased leaf mass per area (LMA), which is a key indicator of leaf construction costs and leaf life span.

## 1. Introduction

As the primary photosynthetic organ in plants, leaves are the major source of reduced carbon skeletons that fuel the biosynthesis of energy-rich macromolecules. However, green leaves in general and protein-rich mesophyll cells in particular, are also a nutritional wellspring for arthropod herbivores and pathogens whose life cycles depend on plants as a food source. Plants effectively counter these biotic threats through constitutive or induced synthesis of myriad defense compounds, as well as development of specialized cells in which these compounds are produced, stored and secreted [1]. The expression of defense traits may exact allocation costs that negatively impact plant growth, particularly when resources such as light and nitrogen are limiting in the environment. Diversion of resources to defense at the expense of construction of new leaf tissue, which otherwise returns revenue in the form of photosynthate over the lifetime of the organ, also imposes so-called opportunity costs that are compounded as plants age [2,3]. These direct costs of defense may also impose ecological costs, including reduced ability to compete with neighboring plants or increased susceptibility to enemies that are not targeted by the induced defense [4,5]. The allocation of limited resources to plant growth and defense has therefore been described as a “dilemma” because growth rates must be sufficient to compete with neighboring plants for light capture while not neglecting investment in defense against plant-eating organisms [3].

Among the biochemical components that must be budgeted between growth and defense are carbon skeletons, ATP and reducing equivalents from photosynthesis, as well as assimilated nitrogen, phosphorous, sulfur, and trace nutrients. Investment of these resources into growth increases the area of resource-acquiring tissues (*i.e.*, photosynthetic structures and roots) and thus accelerates growth capacity. Conversely, investment in defense reduces the ability of herbivores and pathogens to destroy tissues that are needed for resource acquisition. To mitigate the cost of defense on reproductive fitness, plants have evolved inducible defenses, which are deployed upon perception of attack [6]. The plant hormone jasmonate (JA) has become recognized as a chief mediator of inducible defenses. JA is synthesized within minutes of the perception of a threat and exerts transcriptional control over thousands of genes to effect resistance to herbivory and disease [7,8,9].

**Figure 1:**
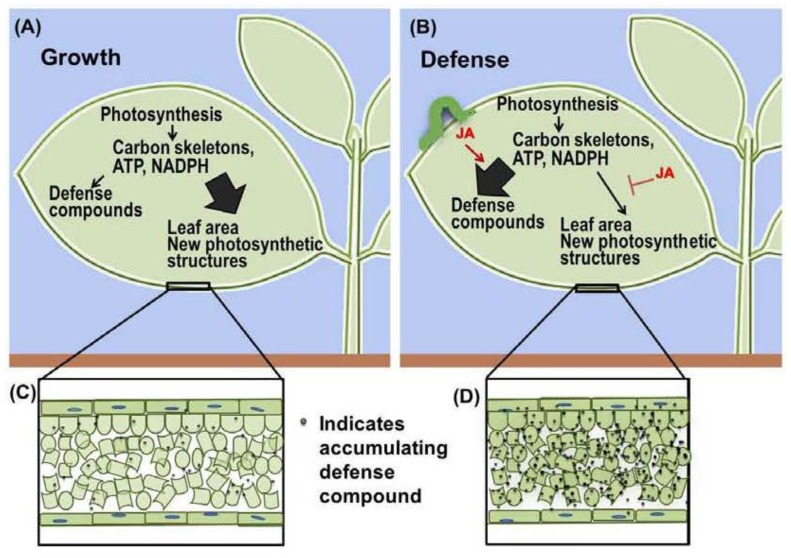
Jasmonate (JA) induces the re-budgeting of resources from tissue expansion to the production of defense compounds. (**A**) Plant growth is achieved using carbon skeletons, ATP, and NADPH from photosynthesis; (**B**) During the JA-mediated defense response, carbon skeletons, ATP, and NADPH that could otherwise contribute to growth are used for *de novo* synthesis of defense compounds; (**C**) In the absence of biotic attack, defense compounds are produced at a low basal level; (**D**) JA triggers the accumulation of defense compounds accompanied by an arrest of tissue expansion. In defended leaf tissue, cell size is similar to that in undefended leaves but the leaf mass per area (LMA) may increase as a consequence of increase carbon deposition into defense compounds.

The expression of defense traits in response to increased JA levels is accompanied by a potent inhibition of growth [10,11]. This has been demonstrated through the use of mutants that lack the ability to produce or perceive JA; such mutants are not only susceptible to biotic aggressors but also show reduced ability to respond to stress cues with growth arrest. In the last decade, much progress has been made toward understanding the core components of JA signaling and the way in which they are integrated into the wider hormone response network [12,13,14]. Despite these advances, however, our understanding of how JA signaling exerts control over growth, either through crosstalk with other growth-signaling pathways or as a consequence of resource allocation to defense (Figure 1), is still in its infancy. In the simplest scenario, carbon and energy diverted from growth would provide for a stoichiometric increase in defense compounds. However, as discussed below, there is a net loss in dry-weight gain within a day or so of the induction of defense signaling, suggesting a more complex scenario. Here, we discuss current opinions regarding the role of JA in governing metabolic tradeoffs between growth and defense, with particular emphasis on how JA modulates resource acquisition and allocation to achieve growth-defense balance.

## 2. Jasmonate Is Central to the Growth-Defense Tradeoff

Jasmonate was first identified more than half a century ago as a component of the essential oil of jasmine flowers [15]. Decades later, it was recognized that JA serves a physiological role in growth inhibition of a wide variety of tissues in both monocot and dicot plants [16,17,18,19,20]. The ability of exogenous JA to inhibit root elongation in a dose-dependent manner is now widely used as a simple quantitative bioassay of JA sensitivity [21,22,23]. A role for JA in defense was recognized by Farmer and Ryan, who demonstrated that airborne JA (JA methyl ester) induces the expression of proteinase inhibitors that contribute to resistance to herbivory [24]. Pioneering work in Zenk’s laboratory further showed that JA is a potent elicitor of defense-related secondary metabolites in suspension-cultured plant cells [25]. Early genetic studies established that JA biosynthesis is essential for insect defense in both tomato and Arabidopsis [26,27]. Subsequently, numerous studies have confirmed the central role of JA in plant defense against a variety of herbivores and necrotrophic pathogens [9,12,28,29].

**Figure 2:**
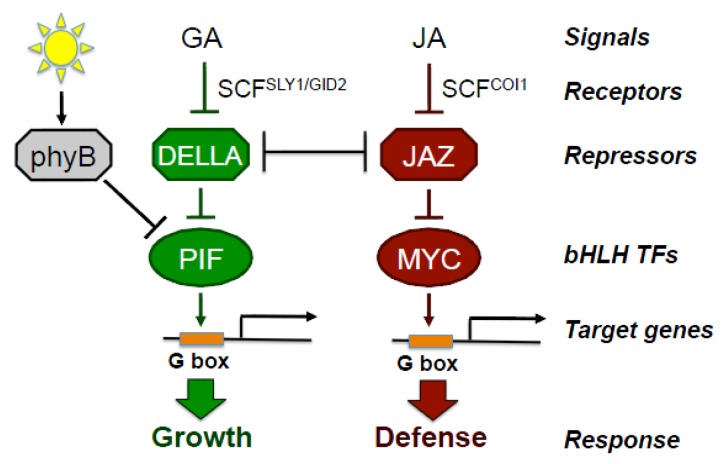
Simplified model depicting interactions between the JA, GA, and phyB signaling pathways. Points of positive and negative regulation are indicated by arrows and perpendicular lines, respectively. bHLH-TFs, basic helix-loop-helix transcription factors that bind G-box *cis*-regulatory elements typically located in the promoter region of response genes. In full sunlight, PIF transcription factors are inhibited by the active conformer of phyB. See text for details.

The dual roles of JA in growth inhibition and defense have mostly been studied as independent phenomena but, in light of the tradeoffs mentioned above, there is a need to understand how these two processes are integrated. Recent work is beginning to uncover mechanisms that tip the growth-defense balance through crosstalk between JA signaling and other hormone response pathways [14,30,31]. A particularly important node of crosstalk is signal antagonism between JA and the growth-promoting gibberellin (GA) pathway (Figure 2). The core JA signaling module includes the JAZ transcriptional repressors that inhibit JA responses by blocking the activity of transcription factors, such as MYC2. The bioactive form of JA, jasmonoyl-l-isoleucine (JA-Ile), promotes the interaction between JAZ proteins and the CORONATINE INSENSITIVE 1 (COI1)-containing Skp1/Cullin/F-box (SCF^COI1^) E3 ubiquitin ligase complex, leading to the ubiquitination and 26S proteasome-dependent degradation of JAZs [32,33,34]. Mutants lacking a functional COI1 are compromised in all known JA-regulated processes and do not respond to the effects of the exogenous hormone [35,36,37]. The JAZ proteins modulate growth responses, at least in part, through direct interaction with the DELLA repressors of the GA pathway (Figure 2). Current evidence indicates that JAZ-DELLA interactions competitively inhibit the ability of each repressor to exert repression on cognate transcription factors [38,39,40,41]. For example, GA-triggered degradation of DELLA proteins by the GID1-GA-SCF^SLY1^ complex frees JAZ and results in greater inhibition of MYC transcription factors and, consequently, repression of defense responses. JAZ-DELLA interactions also favor growth by interfering with the ability of DELLAs to repress PHYTOCHROME-INTERACTING FACTOR (PIF) transcription factors that promote growth responses (Figure 2) [42]. As discussed below, this conserved mechanism of growth-defense balance is modulated by the photoreceptor phytochrome B (phyB) in response to changes in light quality. These collective findings highlight the importance of JAZ-DELLA coupling in transcriptional networks that tune the delicate balance between growth and defense.

In addition to crosstalk between the JA and GA pathways, other hormones have received attention for modulating the effect of JA on growth. Evidence suggests that part of the growth repression arm of JA signaling involves antagonism between JA and the growth-promoting brassinosteroids. A leaky mutation in *DWARF4* (*DWF4*), a gene encoding a key enzyme in brassinosteroid biosynthesis, partially restores the sensitivity of the hypomorphic *coi1-2* allele to the growth inhibitory effects of exogenous JA [43]. Moreover, JA treatment represses *DWF4* transcript accumulation in a *COI1*-dependant manner. Together these results suggest that the growth repression limb of the JA pathway functions in part by blocking brassinosteroid production and downstream activation of cell expansion, a possibility supported by the identification of *DWF4* over-expressing Arabidopsis lines in which root sensitivity to JA is decreased [44]. Growth inhibition by JA may also involve repression of the cytokinin signaling pathway, which is a major positive regulator of cell division activity [17,45].

## 3. Growth Inhibition: Cell Elongation or Cell Division?

Observations of JA-treated plants suggest that JA signaling interferes with processes that promote growth through both cell division and cell elongation. In Arabidopsis leaves, wound-induced JA biosynthesis resulted in a 50% decrease in the calculated number of palisade mesophyll cells per leaf without noticeable effects on the cell size, suggesting that JA inhibits leaf expansion primarily through cell cycle arrest [10]. This finding was supported by decreased activity of a cell-cycle reporter, *CycB1*;*2pro-GUS*, and is consistent with earlier work with tobacco suspension cultured cells in which exogenous JA arrested the cell cycle in the G2 phase [46,47]. An independent study of Arabidopsis leaves at various developmental stages also showed that JA-induced growth inhibition is associated with a strong reduction in cell number. However, delayed cell expansion also contributed to growth inhibition in this experimental system [48].

A number of studies have demonstrated JA-dependent inhibition of Arabidopsis hypocotyl elongation [49,50,51] and rice internode elongation [39]. In studies where cell length was determined, the JA-dependent reduction in cell length could account for differences in internode elongation [39]. Given that extension of Arabidopsis hypocotyls results primarily from cell elongation rather than division [52,53,54], it is likely that hypocotyl growth inhibition by JA is caused by a reduction in cell elongation as well.

Primary root growth inhibition appears to result from a combined reduction in both cell division and cell elongation. In Arabidopsis and rice, exogenous JA inhibits root growth via decreased cell elongation and cell division, although at low JA concentrations decreased cell elongation appears to be primarily responsible for growth inhibition [55,56]. Repeated wounding of Arabidopsis cotyledons triggered a JA-dependent shoot-to-root signal that also reduced root growth by decreasing cell elongation and cell division in the root differentiation zone [57]. Interestingly, loss-of-function mutations in the gene encoding the JA co-repressor NINJA result in reduced cell elongation in the differentiation zone, but not reduced cell division [58]. In summary, JA-dependent growth inhibition occurs through repression of both cell division and elongation, with the relative contribution of each depending on the tissue type.

## 4. Modulation of Carbon Assimilation by Jasmonate

Central to understanding the mechanistic basis of growth–defense tradeoffs is the question of how changes in endogenous JA levels modulate carbon assimilation and subsequent partitioning to various metabolic sinks. The effects of arthropod herbivore attack, simulated herbivory, and exogenous JA on photosynthesis have been examined in a variety of experimental systems, with mixed results. This variability likely reflects differences in damage type and intensity, location and type of tissue damaged, plant developmental stage, and environmental conditions [59]. A preponderance of studies report that treatments associated with activation of JA signaling reduce photosynthesis [59]. One interpretation of downregulation of growth and photosynthetic capacity in herbivore-damaged leaves is that these responses are part of the plant’s defense strategy to limit the availability of food for herbivores [60].

However, robust plant defense responses also depend on a continuous supply of photosynthetic products to fuel local and systemic biosynthesis of defense compounds. Reduction in photosynthetic capacity by silencing of RuBisCO activase in coyote tobacco, for example, attenuated JA-mediated resistance to herbivory [61]. Evidence for positive or compensatory effects further suggests that foliage loss and induced expression of defenses can drive increased photosynthesis in remaining and new leaves, perhaps through altered source-sink relationships [62,63]. These observations are in general agreement with metabolic labeling studies showing that simulated herbivory and exogenous JA stimulate carbon partitioning to belowground tissues [64,65,66]. Zangerl and coworkers [67] reported a positive correlation between herbivore-induced furanocoumarin synthesis and the amount of leaf area having a reduced photosynthetic efficiency, suggesting that the production of this defense compound is relatively insensitive to herbivore-induced reduction in photosynthesis. This study also introduced the use of chlorophyll fluorescence imaging to spatially map the photosynthetic efficiency of whole leaves subjected to herbivory. This approach demonstrated decreases in photosynthesis in damaged tissue at the insect bite zone, which may result from water loss, as well as in undamaged regions of the leaf located a considerable distance from the wound site [59,67].

Downregulation in the expression of photosynthetic genes and, in some cases the corresponding proteins, in response to herbivory or JA treatment was initially observed in barley and since then reported in many other plant species [61,68,69,70,71,72]. Given that photosynthesis-related transcripts and proteins are highly abundant in leaves, this reprogramming of transcription may provide metabolic building blocks needed to redirect the cell’s biosynthetic capacity from growth to defense without short-term negative effects on photosynthesis [70,71]. A recent study by Attaran *et al.* [71] showed that treatment of Arabidopsis with coronatine (COR), a potent agonist of the JA receptor, triggered a strong reduction of photosynthesis-associated gene expression without an accompanying decrease in photochemical efficiency. This experimental system allows for strong and rapid activation of the JA pathway without the complicating effects of tissue damage that accompany herbivory or other wound treatments. In summary, the reported effects of JA on multiple aspects of photosynthesis make it an important consideration for understanding the physiological basis of growth–defense tradeoffs.

## 5. Jasmonate Signaling Promotes Increased Leaf Mass per Area (LMA)

Whereas the effects of JA on photosynthesis have been well-examined, less is understood about how JA modulates the partitioning of fixed carbon to drive various aspects of growth, including leaf area expansion, leaf thickening, petiole elongation, and the construction of new leaves. Modeling of photosynthetic rate and carbon partitioning in relation to growth showed that carbon is partitioned to leaf area expansion and biomass at different rates, and that the relationship between leaf area and biomass is important to consider since leaf area determines light interception [73]. In particular, this study showed that partitioning of carbon to leaf thickness impacts growth more strongly than photosynthesis. Although it is known that activation of the JA defense response reduces leaf expansion [26,71] and biomass accumulation [10,11,49,50], we are not aware of studies that examined how JA affects growth rates of both leaf area and biomass.

**Figure 3:**
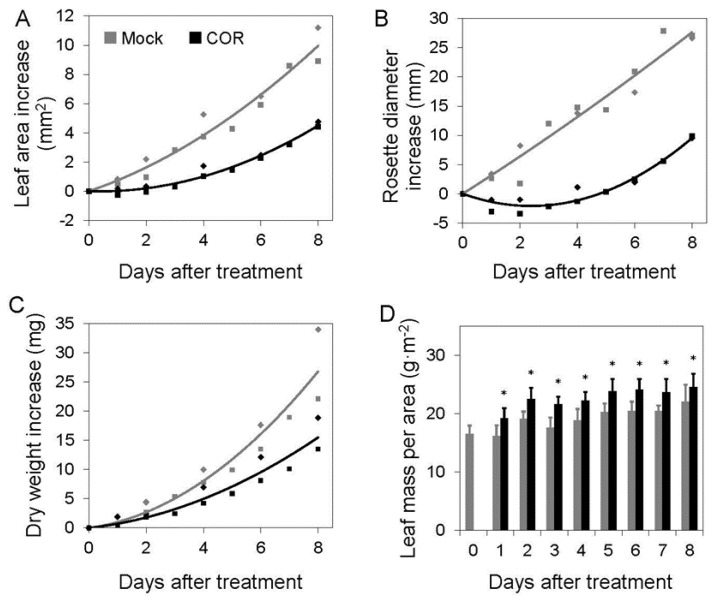
Jasmonate-mediated suppression of leaf area and biomass growth in *Arabidopsis thaliana* plants. Soil-grown Columbia-0 plants treated with mock (grey) or with 5 µM coronatine (COR, black) solutions [71] were measured for leaf area (**A**), rosette diameter (**B**), and dry weight (**C**) at indicated times after treatment. Data are the mean increase in growth (*n* = 12 plants) relative to the day of treatment (day 0) from two independent experiments (diamonds and squares distinguish experiments) and lines are second-order polynomial regressions of combined data from both experiments. Projected leaf area and rosette diameter were determined from overhead images, and dry weight was determined from rosettes (without roots) freeze-dried in a lyophilizer. In (**D**), leaf mass per area (LMA) was calculated from dry weight/leaf area from one experiment in A and C (square points). Error bars are standard deviation and asterisks indicate *P* < 0.05 between mock and COR treatment from a Student’s t-Test.

Our early experiments showed that COR slows leaf area growth almost immediately after treatment (Figure 3A) [71]. COR treatment also arrested rosette diameter expansion (Figure 3B). This rapid effect partly reflects an arrest of petiole elongation and is consistent with the ability of JA to suppress cell elongation [39]. Leaf area and rosette expansion were arrested for several days before increasing again, though at an apparent reduced rate. The resumption in expansion appeared to be due to leaves that emerged after treatment, and which perhaps are less sensitive to the COR treatment. By comparison, dry mass accumulation remained similar to mock-treated plants for at least one day after treatment (Figure 3C). The absence of a corresponding, immediate reduction in dry mass accumulation supports our previous conclusion that activation of JA signaling does not have an immediate repressive effect on photosynthesis in Arabidopsis leaves [71]. These findings are consistent with the idea that reduced leaf area growth in response to JA results in opportunity costs as a consequence of lower total light interception and carbon assimilation on a per plant basis. However, that JA-elicited plants can “pay back” such costs in ways that enhance fitness in the presence of herbivores [74] suggests that a more in-depth analysis of the effects of JA on photosynthetic and non-photosynthetic leaf traits is warranted.

The effects of JA on altered partitioning of resources to leaf area and biomass can be further examined from the ratio of dry mass to leaf area (Leaf Mass per Area, LMA). LMA is useful to describe the resources invested in a given area of leaf and may change as a consequence of variability in leaf thickness, leaf density, or both [75]. LMA has been widely used in studies of plant ecology and agronomy to describe different plant strategies for optimizing fitness to particular environments, as well as for analysis of chemical, physiological, and structural traits that together define the leaf economics spectrum [76]. In general, plants with low LMA tend to acquire resources rapidly and have higher growth rates, whereas plants with high LMA tend to conserve resources and have lower growth rates, but have higher fitness in stressful environments [75]. With their increased resource investment, high-LMA leaves tend to be more durable (e.g., as a consequence of increased thickness), and in terms of defense, are less preferred by herbivores to leaves with low LMA [75]. Thus, it would appear that leaf traits associated with increased LMA have striking similarity to known effects of JA on leaf development and function.

In the experiment described in Figure 3, LMA was significantly increased the day after treatment with COR, and remained significantly higher for the duration of the experiment (Figure 3D). Following day 1, the average difference in LMA between mock- and COR-treated leaves at each time point remained remarkably constant at approximately 3 g/m^2^; this plateau effect may be a consequence of negative feedback loops that rapidly restrain JA responses [9]. One interpretation of the higher LMA of COR-treated leaves is that it reflects a concerted change in strategy from rapid leaf area-based growth to protection of energy acquiring-foliage through investment of resources in leaf defense and structural integrity. Thus, the opportunity costs associated with JA-mediated inhibition of leaf area growth may be counterbalanced by other features of high-LMA leaves, such as increased leaf life span [75]. These areas deserve future attention to better understand the physiological determinants of JA-mediated growth suppression and its relationship to solar energy capture, conversion, and deposition. Studies employing mutants that are impaired in growth-defense balance may provide additional insight into this question. In this context, it is interesting to note that mutants (e.g., *phyB*) exhibiting rapid growth and compromised defense have reduced LMA, whereas mutants with reduced responsiveness to GA grow slowly and have increased LMA [38,75,77]. These observations support the emerging paradigm that crosstalk between the JA and GA signaling pathways plays a significant role in controlling the nexus between leaf growth and defense. Further study of how JA-mediated changes in leaf chemistry are integrated with plasticity in leaf architecture may help to unravel the complexities of growth-defense balance.

## 6. Auditing the Growth-Defense Budget

A comprehensive, quantitative understanding of the metabolic costs associated with partitioning of carbon into growth and defense pathways is still lacking. Efforts to meet this challenge should recognize that the growth-defense dichotomy oversimplifies the complexities of carbon budgeting because many other physiological processes, including reproduction, respiration, exudation, and volatile emission also consume photoassimilates [73]. Proximal analysis provides one approach to estimate the relative cost of producing defense metabolites from precursors in primary metabolism [78,79,80]. Also called detailed biochemical pathway analysis, proximal analysis estimates the energy required to produce biochemical constituents of a tissue, with the sum-total energy requirement for the synthesis, transport and maintenance of all compounds in a tissue equaling the tissue construction cost. This approach requires detailed information about tissue components and the biochemistry of their synthesis. Generally, more reduced secondary metabolites such as terpenoids have a higher metabolic cost than carbohydrate and phenolic metabolites. For example, production of one gram of the monoterpene camphor is calculated to cost 3.1 g glucose equivalents based on the requirements of acetyl-CoA, ATP and NADPH, whereas 1 g of phenol glycoside costs approximately 1.5 to 2 g glucose equivalents [78].

Flux balance analysis may also be used to estimate energy consumption required to deploy induced defenses. Flux balance analysis is performed by constructing, *in silico*, a metabolic network of all of the known or predicted biochemical conversions between compounds within a tissue. The network may be informed by measurements of biochemical activities and/or gene expression/predicted function. Glucosinolate production in Arabidopsis provides an example for which there is sufficient biochemical knowledge to undertake such an analysis [81]. Flux-balance modeling has estimated, for example, that production of glucosinolates in Arabidopsis increases the photosynthetic requirement by 15% [82].

Nitrogen accounts for a relatively small proportion of plant dry weight but is vitally important for plant growth and photosynthesis, and is often a limiting factor for growth in natural environments. Thus, JA-dependent changes in nitrogen assimilation or allocation are expected to have significant impacts on plant growth. Available data indicate that growth–defense tradeoffs involve not only the reallocation of reduced carbon but also substantial redistribution of nitrogen [3,5]. ^15^N flux studies have leveraged nitrogen allocation to RuBisCO and small N-containing defense metabolites as a proxy for growth–defense tradeoffs [83,84]. In young rosette leaves of *Nicotiana attenuata*, simulated herbivory reduced nitrogen investment in RuBisCO and total soluble proteins by 89% [84]. Importantly, nitrogen investment in RuBisCO and total soluble protein was only reduced by 47% in transgenic plants with partially impaired JA biosynthesis. Thus, diversion of nitrogen in response to JA signaling may be a significant driver of growth inhibition.

Calorimetry offers a potentially informative yet simple approach for estimating the biochemical cost of defense. Combustion of plant tissue in a calorimeter gives a simple measure of the total energy stored within the system. Although not as informative as proximal analysis in assessing the contribution of individual compound classes to tissue construction costs, the two methods are typically in broad agreement [85,86]. Moreover, calorimetric estimates can be applied to whole plants. Although this approach has been used to assess altered carbon partitioning to lipid biosynthesis in Arabidopsis leaves [87], to our knowledge the method has not been applied to understanding growth–defense metabolic tradeoffs elicited by JA.

If increased LMA represents a significant component of JA-mediated changes in growth-defense balance as suggested by our preliminary studies (Figure 3D), it may be possible to use this trait as a starting point for quantitative analysis of the growth-defense budget. Variation in LMA as a function of genotype and environment often reflects differences in leaf anatomical traits [75]. Such features include mesophyll tissue volume, cellular volume occupied by the central vacuole and other organelles, volume of air spaces between cells, and leaf thickness. A second and complementary approach to explain variation in LMA is to deconstruct the trait into the chemical constituents of leaf biomass, including structural and non-structural carbohydrates, lignin, proteins, lipids, organic acids, minerals, and secondary metabolites. In-depth knowledge of central carbon metabolism and partitioning in leaves is expected to facilitate this approach [73,88], as is knowledge of how JA, and biotic stress in general, reconfigures primary and secondary metabolism [89].

## 7. Genetic Dissection of Growth-Defense Antagonism

Over the last twenty years, genetic investigations have revealed key steps in the synthesis and perception of JA in a variety of plant species [12,13]. Studies employing these mutants support the general conclusion that stress-induced JA biosynthesis leads to activation of transcriptional modules that simultaneously attenuate vegetative growth and heighten defense against a remarkable spectrum of plant-associated organisms, ranging from microbes to vertebrate herbivores [9]. More recently, molecular genetic investigations are beginning to untangle regulatory circuits through which JA governs growth and defense. The recent identification of mutants that disrupt either the growth or the defense arm of the JA response pathway suggests that the two processes involve interconnected but distinct regulatory networks.

Hu and coworkers [90] used an RNA interference (RNAi)-based screen to identify JAV1 as negative regulator of the defense limb of JA signaling. Silencing of *JAV1* dramatically reduced plant susceptibility to the necrotrophic fungus *Botrytis cinerea* and the lepidopteran herbivore *Spodoptera exigua*. Similar to JAZ repressors, JAV1 was degraded by a pathway that depends both on COI1 and the 26S proteasome. Unlike JAZs, however, JAV1 does not interact directly with COI1 in the presence of COR. The E3 ubiquitin ligase responsible for targeting JAV1 for destruction remains to be identified. Interestingly, *JAV1*-silenced plants had no obvious growth phenotypes despite their enhanced defense response [90]. These findings provide genetic evidence for distinct growth and defense branches of JA response pathway.

Additional evidence that JA-mediated growth and defense responses can be genetically separated comes from recent studies on the interaction between the JA and light signaling pathways that control shade avoidance responses. The shade avoidance syndrome is characterized by rapid growth of stems and petioles, which allows neighboring plants to compete for light [91]. Full sunlight has a high ratio of red (R) to far-red (FR) light, which maintains the phytochrome B (phyB) photoreceptor in its active Pfr form. In this state, phyB represses vegetative growth by inhibiting the activity of PIF transcription factors (Figure 2). Because canopy leaves preferentially absorb red light, light that is transmitted or reflected from neighboring plants has a decreased R:FR ratio. Perception of this shade signal by phyB converts the photoreceptor to its inactive (Pr) form, thereby relieving restraint on PIFs. The ensuing transcriptional responses promote the biosynthesis of auxin, which together with other growth hormones, stimulates cell elongation-type growth [91].

Recent studies have revealed that inactivation of phyB by shade light also suppresses JA-triggered defense responses by a mechanism that involves depletion of DELLA proteins, enhanced stability of JAZ repressors, and accelerated degradation of MYCs [50,77,92,93]. This mechanistic insight into growth-defense balance provides a compelling example of how crosstalk at the DELLA-JAZ interface controls a key aspect of plant phenotypic plasticity, and opens the way to identifying specific regulatory genes that mediate these interactions. *JAZ10*, for example, which encodes several alternative splice variants that strongly repress MYC2 [11,94,95,96], plays a central role in repressing JA-mediated defense responses during the shade avoidance response [50,78,93,94]. Advanced phenotypic profiling techniques have recently been used to establish new genetic connections between the JA and shade avoidance pathways [97].

Although phyB-JA crosstalk appears to be hardwired to ensure mutually exclusive expression of either growth- and defense-related transcriptional programs, there are emerging examples of how genetics can be used to tease apart the physiological basis of these tradeoffs. One example comes from studies with the *sav3* mutant that lacks the ability to synthesize auxin during the shade avoidance response [98]. *sav3* mutant plants lack shade-triggered growth responses but remain sensitive to the attenuating effects of low R:FR on defense responses, indicating that repression of defense does not result simply from diversion of resources to growth [77]. Conversely, a *phyB jaz10* double mutant was shown to retain a constitutive shade avoidance growth response (due to genetic inactivation of phyB) and simultaneously express higher levels of JA-induced secondary metabolites [50]. These collective studies provide clues as to how growth–defense tradeoffs may be genetically manipulated.

## 8. Conclusions and Future Perspectives

Tremendous progress in understanding JA synthesis and signaling in the past 25 years has placed the hormone at the nexus of a complex circuit that controls myriad plant stress responses [9,72]. Now, JA is receiving renewed attention as a growth regulator. The discovery of JAZ-DELLA antagonism has provided deep mechanistic insight into how JA-mediated defense operates in a dynamic balance with growth. Although this and other recent discoveries begin to frame the counterpoise of JA signaling with plant growth, additional effort is required to understand the relationships between JA signaling, deployed defenses, and modulation of growth in the context of leaf expansion, dry-weight gain and partitioning of photoassimilates. Available results indicate that the shift from growth to defense in response to JA signaling is not a simple reallocation of resources but instead involves a rapid and substantial decrease in the rate of dry-matter accumulation. The positive effect of JA signaling on LMA, however, suggests that the hormone may influence leaf architecture via increased leaf thickness and/or leaf density, potentially extending the lifetime photosynthetic output of challenged plants in natural environments. These insights are critically important for an understanding of the survival and fitness of plants in their environment and may reveal opportunities to optimize plant growth, especially in protected agricultural contexts.
